# Experimental Evidence for Diiodohydroxyquinoline-Induced Neurotoxicity: Characterization of Age and Gender as Predisposing Factors

**DOI:** 10.3390/ph15020251

**Published:** 2022-02-19

**Authors:** Ahmed S. Kamel, Ahmed F. Mohamed, Mostafa A. Rabie, Marwa E. Elsherbiny, Kawkab A. Ahmed, Mahmoud M. Khattab, Noha F. Abdelkader

**Affiliations:** 1 Department of Pharmacology and Toxicology, Faculty of Pharmacy, Cairo University, Giza 11562, Egypt; ahmed.seifeldin@pharma.cu.edu.eg (A.S.K.); ahmed.fathi@pharma.cu.edu.eg (A.F.M.); mostafa.mohammed@pharma.cu.edu.eg (M.A.R.); mahmoud.khattab@pharma.cu.edu.eg (M.M.K.); 2 Department of Pharmacology and Toxicology, Faculty of Pharmacy, Ahram Canadian University, 6th of October City 12566, Egypt; marwae@ualberta.ca; 3 Department of Pathology, Faculty of Veterinary Medicine, Cairo University, Giza 12211, Egypt; kawkababdelaziz@yahoo.com

**Keywords:** diiodohydroxyquinoline, neurotoxicity, old/young/male/female rats, behavioral impairment, histopathological abnormalities, principal component analysis

## Abstract

Though quinoline anti-infective agents-associated neurotoxicity has been reported in the early 1970s, it only recently received regulatory recognition. In 2019, the European Medicines Agency enforced strict use for quinoline antibiotics. Thus, the current study evaluates the relation between subacute exposure to diiodohydroxyquinoline (DHQ), a commonly misused amebicide, with the development of motor and sensory abnormalities, highlighting age and gender as possible predisposing factors. Eighty rats were randomly assigned to eight groups according to their gender, age, and drug exposure; namely, four control groups received saline (adult male, adult female, young male, and young female), and the other four groups received DHQ. Young and adult rats received DHQ in doses of 176.7 and 247.4 mg/kg/day, respectively. After 4 weeks, rats were tested for sensory abnormality using analgesiometer, hot plate, and hind paw cold allodynia tests, and for motor function using open field and rotarod tests. Herein, the complex behavioral data were analyzed by principal component analysis to reduce the high number of variables to a lower number of representative factors that extracted components related to sensory, motor, and anxiety-like behavior. Behavioral outcomes were reflected in a histopathological examination of the cerebral cortex, striatum, spinal cord, and sciatic nerve, which revealed degenerative changes as well demyelination. Noteworthy, young female rats were more susceptible to DHQ’s toxicity than their counterparts. Taken together, these findings confirm previous safety concerns regarding quinoline-associated neurotoxicity and provide an impetus to review risk/benefit balance for their use.

## 1. Introduction

8-hydroxyquinoline (8-HQ) is one of the heterocyclic quinolone pharmacophores that possess a wide variety of applications. It has been used in cosmetics as a preservative and a chemical mediator in dye synthesis [1]. Moreover, it possesses antibacterial, antifungal, and antiprotozoal activities [2]. Despite these beneficial effects, signs of toxicity have been reported with its usage. First, liver and spleen hemosiderosis as well as signs of nephrotoxicity have been observed in male rats receiving 8-HQ in their diet [3]. Second, signs of neurotoxicity were reported in several species using halogenated 8-HQ. Noteworthy, 8-HQ produced axonal sheath depletion in sciatic nerves in rats [4]. Clioquinol (iodochlorhydroxyquin) and iodoquinol (diiodohydroxyquinoline; DHQ) are the most known halogenated derivatives for 8-HQ nucleus. Clioquinol was used as intestinal amebicide to treat indigestion and diarrhea but it was withdrawn from the market due to the incidence of subacute myelo-optic neuropathy (SMON) [5]; a syndrome characterized by sensory and motor dysfunction of the lower limb, which has been reported worldwide since early 1970s [6,7]. In addition, a previous study reported that oral clioquinol produced lumbar and sciatic neuronal degeneration to some extent and severe deleterious neuronal degeneration when administered intravenously [4]. Moreover, clioquinol demonstrated cognitive memory impairment in young rats in addition to disturbance in long-term potentiation [8]. However, oral DHQ is still present in the local market and is used as luminal amebicide alone or with metronidazole for amebiasis [9], although it is approved worldwide only as a local antifungal. Moreover, a previous case report in children revealed signs of neurotoxicity associated with DHQ, including seizures and encephalopathy [10]; consequently, the American Academy of Pediatrics banned their usage in children [11]. In addition, another study demonstrated potential mutagenic effects of DHQ in Swiss albino mice [12]. Though quinolone anti-infective agents-related neurotoxicity was reported at least five decades ago, it only recently received regulatory recognition. In 2013, the FDA published a safety alert on mefloquine, a quinoline antimalarial, and added a black box warning to its label denoting neurologic adverse effects [13]. In 2019, the European Medicines Agency enforced suspension and/or restriction of use for quinolone and fluoroquinolone antibiotics (EMA/175398/2019) [14]. Consequently, the question was raised again regarding DHQ neurotoxic effect versus its beneficial role. Three factors were considered in this study, including age, sex, and treatment. Age was selected, as signs of toxicity are variable among young and adult rats, due to different metabolic and excretory pathways of the drugs. Gender was also chosen, as the hormone level, estrogen, in females, affects the metabolism of drugs; hence the toxicity is gender-dependent. Thus, the present study was designed to characterize DHQ neurotoxic effects in both young and adult Wistar rats of both sexes. A battery of behavioral experiments for motor, sensory, and anxiety was used. Furthermore, the involvement of central and peripheral nervous systems was investigated using histopathological examination of brain, spinal cord, and sciatic nerve tissues. Results were then discussed considering the extensive neurotoxicity of various anti-infective quinolines reported many decades ago, but insufficiently recognized and regulated.

## 2. Results

### 2.1. Motor Impairments Instigated by Diiodohydroxyquinoline Administration in Young/Adult Male/Female Rats

**Figure 1:**
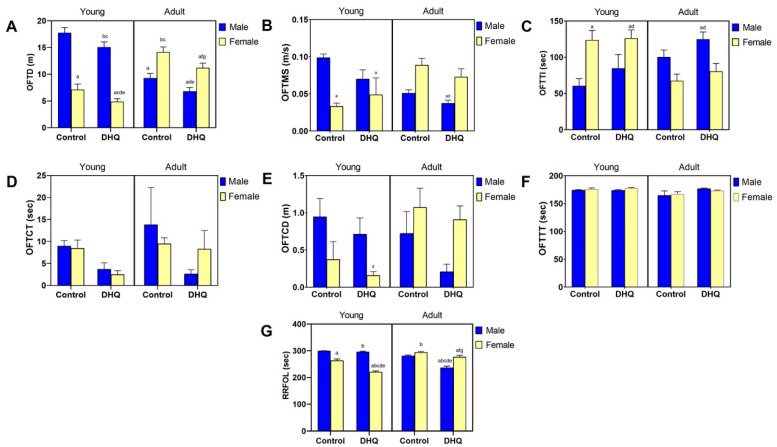
Motor impairments instigated by diiodohydroxyquinoline administration in young/adult male/female rats. Panels represent: (**A**) distance traveled (OFTD), (**B**) mean speed (OFTMS), (**C**) time immobile (OFTTI), (**D**) central time (OFTCT), (**E**) central distance (OFTCT), and (**F**) thigmotaxis time (OFTTT) in the open field test as well as (**G**) fall off latency (RRFOL) in the rotarod test. Each bar with a vertical line represents the mean ± S.D. of 10 rats per group. a vs. control young male, b vs. control young female, c vs. control adult male, d vs. control adult female, e vs. DHQ young male, f vs. DHQ young female, and g vs. DHQ adult male using three-way ANOVA followed by Tukey’s post hoc test; *p* < 0.05. DHQ; diiodohydroxyquinoline.

Sub-acute administration of DHQ produced neuropathic changes in rats that were manifested as both motor and sensory abnormalities. Figure 1A–C,G shows that DHQ resulted in a significant decrease in OFTD (84.9%, 68.2%, 73.2%, and 79.2%) as well as RRFOL (98.8%, 83.8%, 84.1%, and 94.1%) for treated young males, young females, adult males, and adult females, respectively, as compared to their control counterparts. The change in RRFOL was both age- and gender-dependent (Treatment × Age × Gender interaction, *p* < 0.0001). It is noteworthy that neither OFTMS nor OFTTI were significantly affected by DHQ treatment. This was paralleled by a reduction in OFTCT as a result of DHQ administration (40.9%, 29.3%, 18.8%, and 87.4%), reflecting a possible anxiogenic effect of DHQ. No statistical significance, however, was witnessed in OFTCD or OFTTT due to DHQ administration (Figure 1D–F).

### 2.2. Sensory Impairments Instigated by Diiodohydroxyquinoline Administration in Young/Adult Male/Female Rats

**Figure 2:**
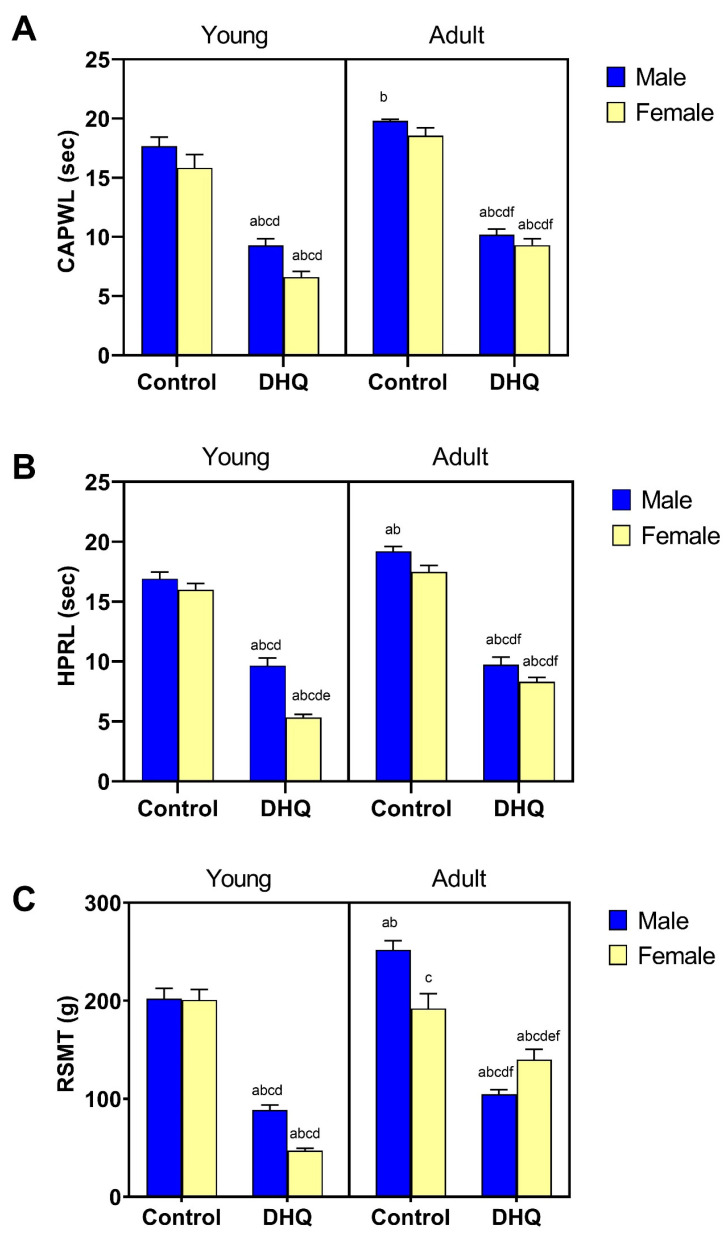
Sensory impairments instigated by diiodohydroxyquinoline administration in young/adult male/female rats. Panels represent: (**A**) paw withdrawal latency (CAPWL) in the cold allodynia test, (**B**) reaction latency (HPRL) in the hot plate test, and (**C**) mechanical threshold (RSMT) in Randall–Selitto test. Each bar with a vertical line represents the mean ± S.D. of 10 rats per group. a vs. control young male, b vs. control young female, c vs. control adult male, d vs. control adult female, e vs. DHQ young male, and f vs. DHQ young female using three-way ANOVA followed by Tukey’s post hoc test; *p* < 0.05. DHQ; diiodohydroxyquinoline.

Sensory anomalies resulting from DHQ administration are depicted in Figure 2. As shown in Figure 2A,B, rats that were administered DHQ had markedly lowered reaction latencies in the hind paw cold allodynia (52.6%, 41.7%, 51.4%, and 50.1%) as well as in the hot plate test (56.9%, 33.3%, 50.7%, and 47.5%) in young males, young females, adult males, and adult females. These changes were more evident in young female rats (Treatment × Age × Gender, *p* = 0.015 “hot plate”). Figure 2C illustrates the mechanical hyperalgesia associated with DHQ administration that was evidenced by a significant reduction in mechanical threshold in the Randall–Selitto test (43.8%, 23.4%, 41.5%, and 72.8%) in young males, young females, adult males, and adult females, respectively. Consistent with findings from the hind paw cold allodynia test, mechanical hyperalgesia was more evident in young females (Treatment × Age × Gender, *p* < 0.0001).

### 2.3. Linear Regression and Principal Component Analysis

**Figure 3:**
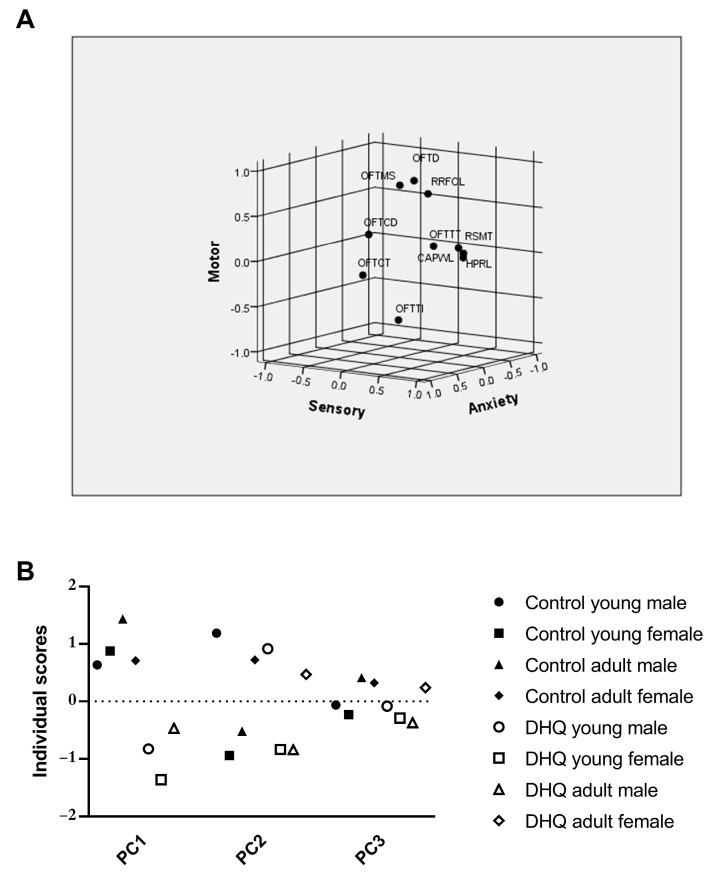
Diagram of the first three components of principal component analysis in rotated space (**A**) and principal component scores of rats in each group derived from PCA of behavioral variables (**B**). Data points represent the mean score of each group. Scores for each component were compared using MANOVA; *p* < 0.05. DHQ; diiodohydroxyquinoline, OFT; open field test, OFTD; distance traveled, OFTMS; mean speed, OFTTI; time immobile, OFTCT; central time, OFTCT; central distance, OFTTT; thigmotaxis time, RRFOL; rotarod test fall off latency, CAPWL; cold allodynia paw withdrawal latency, HPRL; hot plate test reaction latency, RSMT; Randall–Selitto mechanical threshold.

The correlations among variable behavioral traits associated with DHQ administration were illustrated using two-tailed Pearson’s correlation matrix (Table 1). OFTD correlated positively with all sensory measures, namely CAPWL, HPRL, and RSMT. Similar correlations with sensory measures were observed with RRFOL and OFTCT. OFTTT correlated negatively with most sensory measures as well as RRFOL. Other measures within the tests were necessarily correlated. Principal component analysis (PCA) was then performed, which produced three factors with eigenvalues greater than 1. These three factors account for 78.5% of the total variation in the correlation matrix; varimax rotation was performed on them. A graphical representation of the first three components and the Euclidean distances of variables is provided in Figure 3A. Component patterns are provided in Table 2. PCI explained 29.1% of variation (after rotation) and was highly positively loaded (factor loading> 0.5) by CAPWL, HPRL, and RSMT, hence this component was considered to reflect sensory function. PCII explained 27.4% of the variation and was positively loaded by OFTD, OFTMS, and RRFOL, whereas OFTTI was highly negatively loaded on this component, suggesting that PCII is related to the rats’ motor ability. For PCIII, which explained 22% of total variation, positively loading behavioral traits were OFTCT and OFTCD, while OFTTT was negatively loaded; thus, PCIII represents anxiety-related behavior. Finally, PC scores for individual rats in each group were compared using MANOVA (Figure 3B). An analysis of PCI revealed that DHQ significantly affected sensory function in rats (*p* < 0.0001) and that this effect was age- and gender-dependent (Treatment × Age × Gender interaction, *p* = 0.0002). Interestingly, MANOVA analysis of PCII scores demonstrated no significant effect of DHQ on the overall motor ability of rats, although the treatment markedly affected some individual variables that loaded highly on this component. Similarly, the analysis of PCIII scores demonstrated no significant difference in anxiety-like behaviors due to DHQ treatment.

### 2.4. Histopathological Alterations Instigated by Diiodohydroxyquinoline Administration in Cerebral Cortices of Young/Adult Male/Female Rats

**Figure 4:**
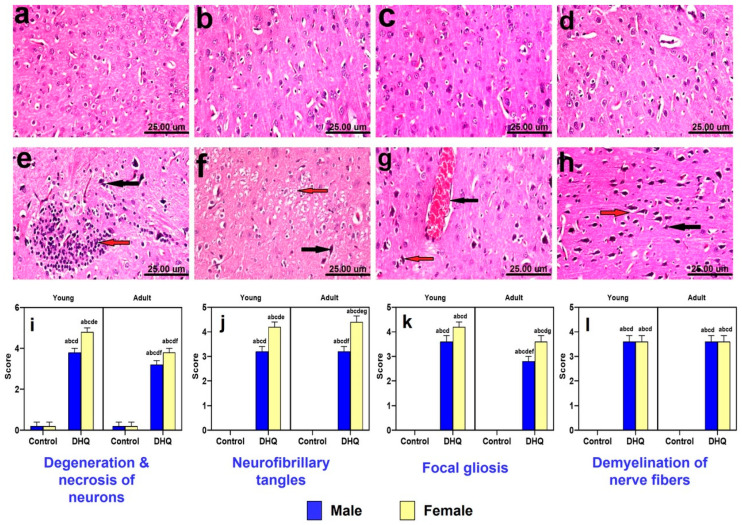
Photomicrograph of hematoxylin and eosin-stained cerebral cortices tissue sections of: (**a**–**d**) control rats, (**a**) young male, (**b**) young female, (**c**) adult male, and (**d**) adult female, showing normal histological picture. (**e**) DHQ young male showing shrunken necrotic neurons with pyknotic nuclei (black arrow) and focal gliosis (red arrow). (**f**) DHQ young female showing necrosis of neurons (black arrow) and demyelination of nerve fibers (red arrow). (**g**) DHQ adult male showing congestion of cerebral blood vessels (black arrow) and necrosis of neurons with neuronophagia (red arrow). (**h**) DHQ adult female showing massive neuronal degeneration (black arrow) with formation of neurofibrillary tangles (red arrow) (scale bar 25 um). Graphical representation of histopathological scores for (**i**) degeneration and necrosis of neurons, (**j**) neuro fibrillary tangles, (**k**) focal gliosis, and (**l**) demyelination of nerve fibers. Each bar with a vertical line represents the mean ± S.D. of 5 rats per group. a vs. control young male, b vs. control young female, c vs. control adult male, d vs. control adult female, e vs. treated young male, f vs. treated young female, and g vs. treated adult male using three-way ANOVA followed by Tukey’s post hoc test; *p* < 0.05. DHQ; diiodohydroxyquinoline.

Microscopically, cerebral cortices of control animals (young males, young females, adult males, and adult females) revealed normal histological structure (Figure 4a–d). On the contrary, treated young males showed neuropathic alterations as shrunken necrotic neurons with pyknotic nuclei, focal gliosis (Figure 4e), and neuronophagia of degenerated neurons. Additionally, examined cortices of treated young female rats revealed massive necrosis of neurons, demyelination of nerve fibers (Figure 4f) meningeal congestion, and focal gliosis. Moreover, cerebral cortices of treated adult male rats showed vascular congestion of cerebral blood vessels, necrosis, and pyknosis of neurons associated with neuronophagia (Figure 4g). Marked neuropathic alterations were observed in cortices of treated adult females, described as massive neuronal degeneration with formation of neurofibrillary tangles (Figure 4h), neuronophagia, focal gliosis, and demyelination of nerve fibers. The statistical analysis of histopathological scores for different indices of cortical injury (Figure 4i–l) demonstrated marked degeneration, neurofibrillary tangles, focal gliosis as well as nerve fiber demyelination instigated by DHQ treatment. Most of these effects were largely gender dependent.

### 2.5. Histopathological Alterations Instigated by Diiodohydroxyquinoline Administration in Striatum of Young/Adult Male/Female Rats

**Figure 5:**
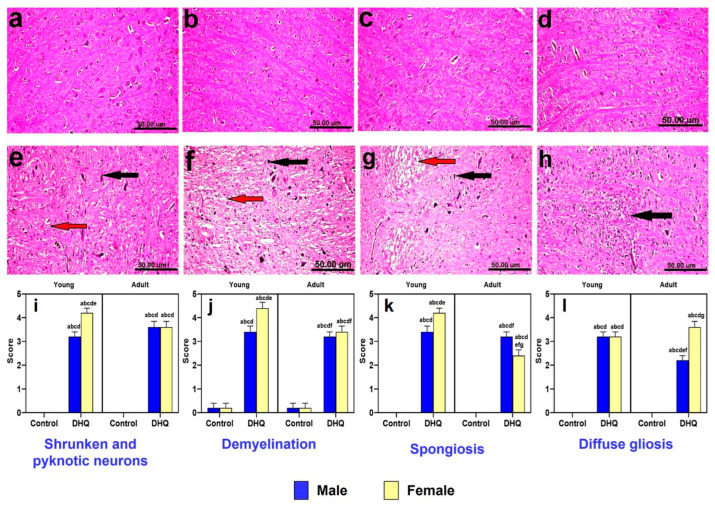
Photomicrograph of hematoxylin and eosin-stained striatum tissue sections of: (**a**–**d**) control rats (**a**) young male, (**b**) young female, (**c**) adult male, and (**d**) adult female, showing the normal histological picture. (**e**) DHQ young male showing shrunken, atrophied and pyknotic neurons (black arrow) and demyelination (red arrow). (**f**) DHQ young female showing necrosis of neurons (black arrow) and severe demyelination with spongiosis of striatum (red arrow). (**g**) DHQ adult male showing necrosis of neurons (black arrow) and severe demyelination (red arrow). (**h**) DHQ adult female showing diffuse gliosis (black arrow) (scale bar 50 um). Graphical representation of histopathological scores for (**i**) shrunken and pyknotic neurons, (**j**) demyelination, (**k**) spongiosis, and (**l**) diffuse gliosis. Each bar with a vertical line represents the mean ± S.D. of 5 rats per group. a vs. control young male, b vs. control young female, c vs. control adult male, d vs. control adult female, e vs. treated young male, f vs. treated young female, and g vs. treated adult male using three-way ANOVA followed by Tukey’s post hoc test; *p* < 0.05. DHQ; diiodohydroxyquinoline.

Striatum of control animals (young males, young females, adult males, and adult females) revealed normal histological picture (Figure 5a–d). On the other hand, all treated groups demonstrated more or less similar histopathological alterations, which varied in severity. The changes were more severe in female groups than male ones. The neuropathic changes were confined as shrunken, atrophied, and pyknotic neurons associated with severe demyelination and spongiosis (Figure 5e–g). Diffuse gliosis was also noticed in sections from treated adult female rats (Figure 5h). Lesion scores demonstrate significant pyknosis, spongiosis, gliosis, and demyelination induced by DHQ, with most of these effects being both age and gender dependent (Figure 5i–l).

### 2.6. Histopathological Alterations Instigated by Diiodohydroxyquinoline Administration in Spinal Cords of Young/Adult Male/Female Rats

**Figure 6:**
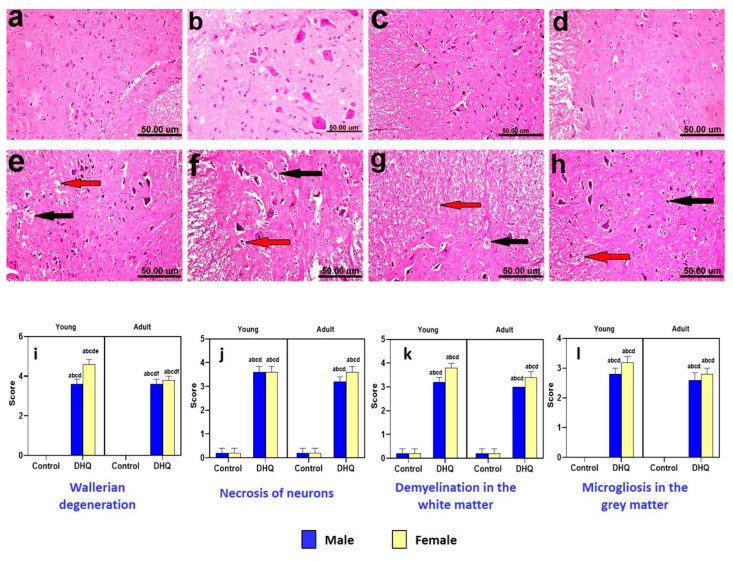
Histological hematoxylin and eosin-stained sections from spinal cords of (**a**–**d**) control rats, (**a**) young male, (**b**) young female, (**c**) adult male, and (**d**) adult female, showing the normal histological structure. (**e**) DHQ young male showing Wallerian degeneration (black arrow), demyelination (red arrow), and diffuse microgliosis in the gray matter. (**f**) DHQ young female showing marked Wallerian degeneration (black arrows), necrosis, and karyorrhectic nuclear changes with microgliosis (red arrow) and central chromatolysis of motor neurons (arrowhead). (**g**) DHQ adult male showing Wallerian degeneration (black arrow) and severe demyelination (red arrow) in the white matter. (**h**) DHQ adult female showing necrosis of neurons with diffuse gliosis (black arrow) and demyelination (red arrow) (scale bar 50 um). Graphical representation of histopathological scores for (**i**) Wallerian degeneration, (**j**) necrosis of neurons, (**k**) demyelination in the white matter, and (**l**) microgliosis in the gray matter. Each bar with a vertical line represents the mean ± S.D. of 5 rats per group. a vs. control young male, b vs. control young female, c vs. control adult male, d vs. control adult female, e vs. treated young male, and f vs. treated young female using three-way ANOVA followed by Tukey’s post hoc test; *p* < 0.05. DHQ; diiodohydroxyquinoline.

Histological H&E-stained sections from spinal cords of all control groups revealed normal histological structure (Figure 6a–d). Meanwhile, the treated male rats (young and adult) showed Wallerian degeneration, demyelination in the white matter, and diffuse microgliosis in gray matter (Figure 6e,g). Examined sections from female groups revealed more severe lesions than male groups. Spinal cords of treated young females showed marked Wallerian degeneration, necrosis, and karyorrhectic nuclear changes with microgliosis and central chromatolysis of motor neurons (Figure 6f). Moreover, the treated adult females showed necrosis of neurons with diffuse gliosis and demyelination (Figure 6h). Figure 6i–l illustrates marked Wallerian degeneration, necrosis, white matter demyelination, and gray matter microgliosis induced by DHQ.

### 2.7. Histopathological Alterations Instigated by Diiodohydroxyquinoline Administration in Sciatic Nerves of Young/Adult Male/Female Rats

**Figure 7:**
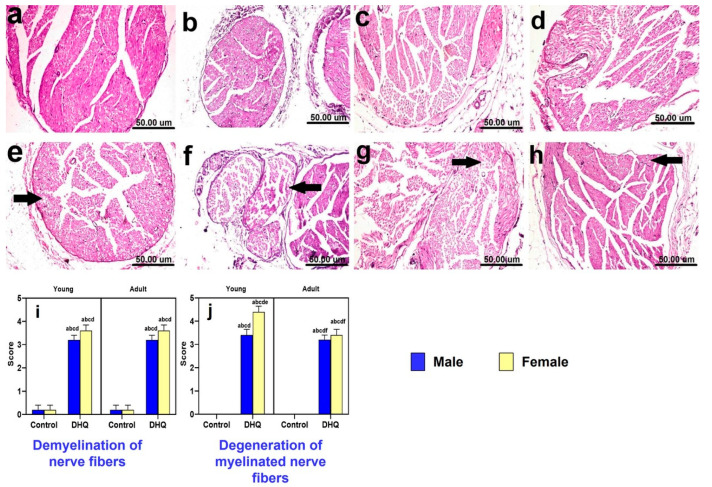
Histological hematoxylin and eosin-stained sections from sciatic nerves of (**a**–**d**) control rats, (**a**) young male, (**b**) young female, (**c**) adult male, and (**d**) adult female, showing the normal histological architecture of nerve fascicles containing nerve fibers and surrounded by perineurium connective tissue. (**e**) DHQ young male showing demyelination of nerve fibers (black arrow). (**f**) DHQ young female showing marked Wallerian demyelination of nerve fibers (black arrows) and dark degenerated myelinated nerve fibers. (**g**) DHQ adult male showing demyelination of nerve fibers (black arrow). (**h**) DHQ adult female showing demyelination of nerve fibers (black arrow) (scale bar 50 um). Graphical representation of histopathological scores for (**i**) demyelination of nerve fibers and (**j**) degeneration of myelinated nerve fibers. Each bar with a vertical line represents the mean ± S.D. of 5 rats per group. a vs. control young male, b vs. control young female, c vs. control adult male, d vs. control adult female, e vs. treated young male, and f vs. treated young female using three-way ANOVA followed by Tukey’s post hoc test; *p* < 0.05. DHQ; diiodohydroxyquinoline.

Histological H&E-stained sections from sciatic nerves of all control groups showed normal histological architecture of nerve fascicles containing nerve fibers and surrounded by perineurium connective tissue (Figure 7a–d). On the other hand, all treated groups showed more or less similar histopathological alterations described as demyelination of nerve fibers, marked Wallerian demyelination of nerve fibers, and dark degenerated myelinated nerve fibers (Figure 7e–h). Figure 7i,j illustrates the lesion scores for demyelination and neuronal degeneration in sciatic nerve in different experimental groups. DHQ treatment induced a marked deterioration of both indices.

### 2.8. Myelin Alterations Instigated by Diiodohydroxyquinoline Administration in Cortex, Striatum, Spinal Cords, and Sciatic Nerves of Young/Adult Male/Female Rats

**Figure 8:**
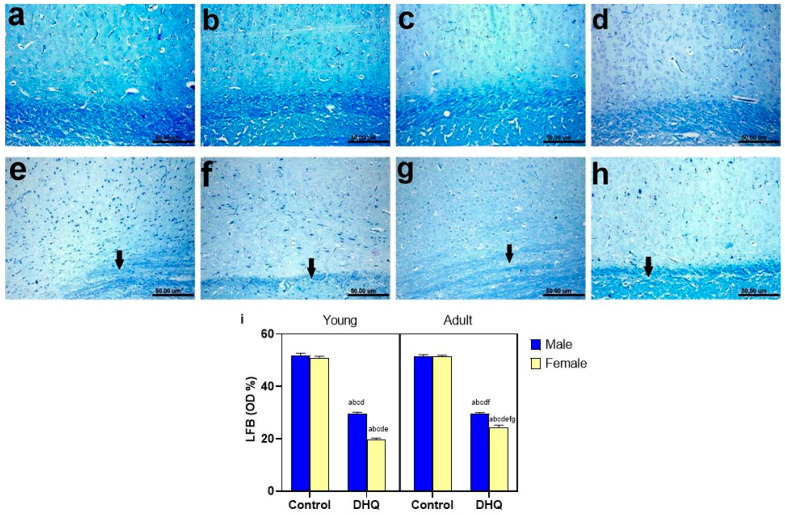
Histological Luxol fast blue-stained sections from cerebral cortex of (**a**–**d**) control rats, (**a**) young male, (**b**) young female, (**c**) adult male, and (**d**) adult female, showing apparently normal dark blue myelinated axons. (**e–h**) show demyelination (black arrow), (**e**) DHQ young male, (**f**) DHQ young female, (**g**) DHQ adult male, and (**h**) DHQ adult female. (Scale bar 50 um). (**i**) Quantitative analysis of LFB staining in cortical sections. Each bar with a vertical line represents the mean ± S.D. of 5 rats per group (5 fields/rat). a vs. control young male, b vs. control young female, c vs. control adult male, d vs. control adult female, e vs. DHQ young male, f vs. DHQ young female, and g vs. DHQ adult male using three-way ANOVA followed by Tukey’s post hoc test; *p* < 0.05. DHQ; diiodohydroxyquinoline.

**Figure 9:**
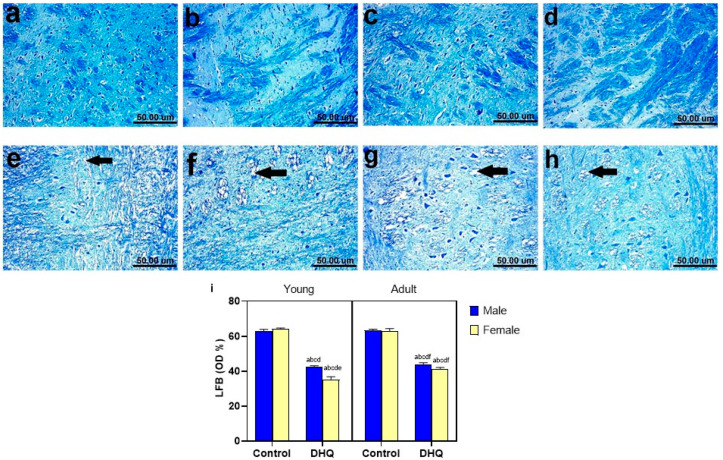
Histological Luxol fast blue-stained sections from striatum of (**a**–**d**) control rats, (**a**) young male, (**b**) young female, (**c**) adult male, and (**d**) adult female, showing apparently normal dark blue myelinated axons. (**e**–**h**) showing demyelination (black arrow), (**e**) DHQ young male, (**f**) DHQ young female, (**g**) DHQ adult male, and (**h**) DHQ adult female. (Scale bar 50 um). (**i**) Quantitative analysis of LFB staining in striatal sections. Each bar with a vertical line represents the mean ± S.D. of 5 rats per group (5 fields/rat). a vs. control young male, b vs. control young female, c vs. control adult male, d vs. control adult female, e vs. DHQ young male, and f vs. DHQ young female, using three-way ANOVA followed by Tukey’s post hoc test; *p* < 0.05. DHQ; diiodohydroxyquinoline.

**Figure 10:**
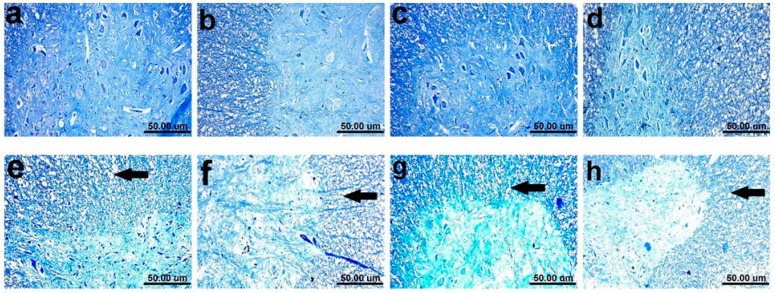

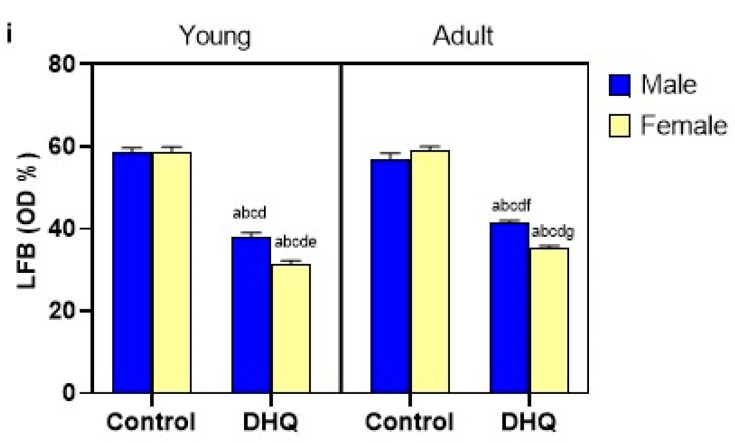
Histological Luxol fast blue-stained sections from spinal cords of (**a**–**d**) control rats, (**a**) young male, (**b**) young female, (**c**) adult male, and (**d**) adult female, showing apparently normal dark blue myelinated axons. (**e**–**h**) showing demyelination in white and gray matter (black arrow), (**e**) DHQ young male, (**f**) DHQ young female, (**g**) DHQ adult male, and (**h**) DHQ adult female. (Scale bar 50 um). (**i**) Quantitative analysis of LFB staining in sections from spinal cords. Each bar with a vertical line represents the mean ± S.D. of 5 rats per group (5 fields/rat). a vs. control young male, b vs. control young female, c vs. control adult male, d vs. control adult female, e vs. DHQ young male, f vs. DHQ young female, and g vs. DHQ adult male using three-way ANOVA followed by Tukey’s post hoc test; *p* < 0.05. DHQ; diiodohydroxyquinoline.

**Figure 11:**
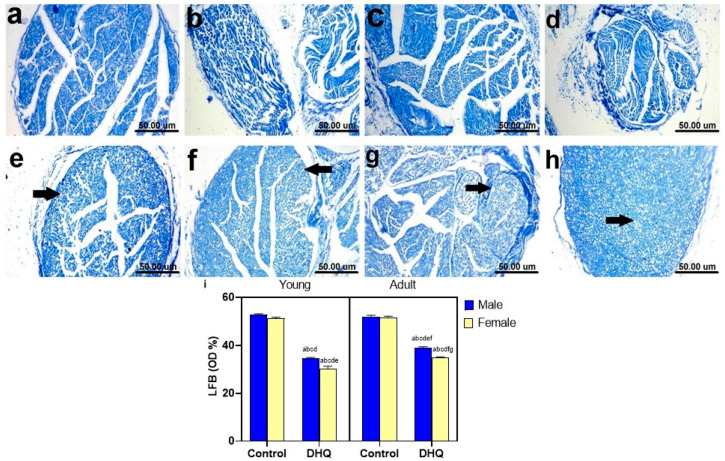
Histological Luxol fast blue-stained sections from sciatic nerves of (**a**–**d**) control rats, (**a**) young male, (**b**) young female, (**c**) adult male, and (**d**) adult female, showing apparently normal dark blue myelinated axons. (**e–h**) showing demyelination of nerve fibers (black arrow), (**e**) DHQ young male, (**f**) DHQ young female, (**g**) DHQ adult male, and (**h**) DHQ adult female. (Scale bar 50 um). (**i**) Quantitative analysis of LFB staining in sections from sciatic nerves. Each bar with a vertical line represents the mean ± S.D. of 5 rats per group (5 fields/rat). a vs. control young male, b vs. control young female, c vs. control adult male, d vs. control adult female, e vs. DHQ young male, f vs. DHQ young female, and g vs. DHQ adult male using three-way ANOVA followed by Tukey’s post hoc test; *p* < 0.05. DHQ; diiodohydroxyquinoline.

Normal dark blue myelinated axons were observed in histological LFB-stained sections from cortex, striatum, spinal cords, and sciatic nerves of all control groups (Figure 8a–d, Figure 9a–d, Figure 10a–d and Figure 11a–d). In contrast, demyelination with faint blue-stained axons was demonstrated in the examined sections (striata, spinal cords, and sciatic nerves) from all treated groups (Figure 8e–h, Figure 9e–h, Figure 10e–h and Figure 11e–h).

## 3. Discussion

The previously reported tendency of clioquinol (one representative of 8-HQ) to affect sensorimotor function prompted us to assess the effect of DHQ on motor performance and pain-like behavior in rats. Pain-related behaviors were evaluated using different stimuli, such as hind paw cold allodynia, hot plate thermal algesia, and mechanical hyperalgesia. These tests can assess and quantify pain-like behaviors as well as detect any variability in pain-threshold. Surprisingly, DHQ-treated rats demonstrated allodynia and hyperalgesia responses with lowered pain threshold in the three aforementioned tests compared to control groups; this was especially prominent in the young female group. This was observed in clioquinol-treated mice and was rationalized by the ability of clioquinol to chelate zinc and copper ions, as this chelation enhances activation of transient receptor potential ankyrin 1 (TRPA1) in sensory neurons [15]. TRPA1 has been proposed as a sensory transduction molecule for both cold and mechanical stimuli and any increase in TRPA1 is associated with hyperalgesia. The severity of histopathological alterations was more pronounced in female groups, especially those of young age. The female gender may be a predisposing factor to the neurotoxicity of DHQ, as reported in the present study by a lower threshold of tactile and pain sensation as well as in previous clinical cases subjected to clioquinol [16,17]. Specific scores of histopathological lesions for cortex, striatum, spinal cord, and sciatic nerve in diiodohydroxyquinoline-treated animals are presented in supplementary Table S1.

There was a coincidence between heat, cold, and mechanical withdrawal responses, as indicated by Pearson’s correlation matrix, which attracts attention to the involvement of the sensory element that is common to these three stimuli in DHQ-induced neurotoxicity. In addition, while conducting this study, a gross inspection of the hind paw revealed no evidence of erythema or tissue damage after mechanical and thermal testing. This turned the sight to histopathological examinations. The present study demonstrates demyelination in various investigated areas/organs, as confirmed by LFB-stain, and this shed light on the demyelinating side effects of DHQ. Demyelination disrupts rates of nerve impulse transmission and creates abnormal sensory phenomena, such as allodynia, hyperalgesia, and spontaneous pain. Abnormal sensory phenomena are associated with human peripheral demyelinating neuropathies, such as Charcot–Marie–Tooth disease and Guillain-Barré syndrome. The damage to sensory nerves as a result of peripheral demyelinating disease has been linked to pain and heightened sensitivity to touch [18,19]. This was also observed with clioquinol, which causes symmetrical demyelination in lateral and posterior funiculi of the spinal cord in humans [20]. It was demonstrated that clioquinol lowers vitamin B12 in the brain of mice and this rationalized the involvement of hydroxyquinoline derivatives, including DHQ in demyelination and neuropathy [21]. Ultimately, this is strong evidence that sensory fiber dysfunction is a significant feature of DHQ intoxication.

The PCA is a data analytical, rather than statistical, procedure. The present study utilized the privilege of using PCA for classification of a highly complex bundle of variables of different behavioral tests, which can be reduced to only countable components that mirror the behavioral features assessed by these variables. Factor analysis has been used previously to explore the relation between behavioral tests [22] as well to detect sex- and age-specific behavior [23,24] and eventually to demonstrate the most prominent effect of the drug within several altered behavioral variables [25]. The Kaiser rule allowed us to choose components with eigenvalues of at least one concomitantly with the scree plot (data not shown) that resulted in the projection of three components that represent 78.5% of the total variation. Varimax rotation maximized variance to increase the squared correlation of items related to one factor and on the other hand decrease the correlation on other factors. In the current study, PCA allowed us to categorize variables of sensory and motor tests into three components. The analysis produced PCI, which explained 29.1% of variation and was mainly composed of sensory-related variables. Analysis of PCI revealed that DHQ significantly affected sensory function in rats; this effect was age- and gender-dependent, where DHQ-treated rats exhibited lower PCI scores than untreated rats, especially female young rats. This finding was documented by the American Academy of Pediatrics Committee on Drugs and cautioned against iodoquinol use in children and infants due to neurotoxicity [11] and it matches findings from clinical reports of hydroxyquinoline products [17]. Obviously, it was found that sensory disturbances were profound in clioquinol-deteriorated patients in Japan, reaching 97% of mainly feminine cases [16], which is in line with the present findings of PCA.

Regarding motor performance, rotarod and open field tests were utilized to analyze coordination and balance. Motor disturbance is most often determined by the rotarod test. Noteworthy, performance in the rotarod task depends mainly on functionality of the nigrostriatal dopamine system [26]. The findings of rotarod demonstrated a clear deficit in DHQ-treated rats, as they have shorter fall off latency compared to normal controls. This is in agreement with histopathological studies, which demonstrated neuropathic changes in the nigrostriatal pathway confined as shrunken, atrophied, and pyknotic neurons associated with severe demyelination and spongiosis. The severity of histopathological alterations was more pronounced in female groups’, especially the young age. This was consistent with results from a previous study, where a halogenated quinolinol, clioquinol, caused marked sensory and motor disturbance in the lower extremities in Japan [27,28]. Taken together, the present study utilized the rotarod accelerating protocol, as it is considered a more discriminative test that correlates motor deficits against lesion size [29] and this was elucidated herein where the most deteriorated gender, the female, showed significant lesions in the striatum and sciatic nerve concomitantly with increased falling rates of rotarod compared with the counterparts.

The lack of direct evidence of specific motor disturbance, together with DHQ’s demonstrated degenerative effects in the animal model in the striatum and sciatic nerve, suggests that such impaired behavior, analogous to that previously observed with clioquinol, may set a more plausible pathophysiological illustration for these symptoms. Herein, PCII explained 27.4% of variation and was related to the rats’ motor performance. This component of motor function is unrelated to sensory function, as PCII is orthogonal to sensory-related PCI. Sensory-related variables contributed to a much lesser extent to PCII. The variables, OFTD, OFTMS, OFFTTI and RROFL-loaded PCII, with the most affected by DHQ administration, OFTD and RROFL. However, RROFL demonstrated positive correlation with withdrawal threshold in the three sensory tasks. To our knowledge, alteration in the sensory function is accompanied, accordingly, with alteration in proprioception, indicating motor deficits [30]. Muscle proprioceptors encode information and transmit it to networks in the spinal cord to coordinate and adapt muscles according to posture and gait. Thus, the motor abnormality in the current study may be in part secondary to sensory dysfunction. It seems that the outcome of PCA is in line with the previous rationale that sensory-related variables loaded profoundly PCI and captured 29.1% of total variance followed by motor variables that loaded PCII. However, this study applies the profound difference of PCA in respect to linear regression analysis. However, there were correlations between the performance in different tasks, but generally, PCA bundles within-test variables and discriminates between-test variables more effectively than regression analysis [22]. Therefore, the motor disturbance may be a direct effect of DHQ on spinal cord and striatum as presently witnessed by histopathological aberrations rather than as secondary to sensory deficits. PCA demonstrated this speculation that to a less extent the motor function is not the major deteriorated domain by DHQ, such as the sensory function.

The open field test assesses locomotor and exploratory activity, which can be correlated with locomotive function and neuromuscular disease [31,32,33,34]. In this regard, DHQ significantly affected locomotion activity in the open field arena, as evidenced by the reduced total distance covered by rats, which confirms the impairment of motor function, as previously recorded in the rotarod. This is in line with a comparative study that documented an abnormality in locomotion in patients treated by clioquinol that lasted for 32 years, where complete loss of locomotion was reached [16]. Biochemically, metal chelation with zinc makes hydroxyquinoline derivatives more lipophilic and consequently more bioavailable, affecting the central nervous system [5]. This mechanism could explain the neurotoxic effect of DHQ, a clioquinol analogue, as well as the alteration of muscle coordination and motor performance. Indeed, there is a positive forward relationship between motor dysfunction and CNS demyelination: as the extent of CNS demyelination increases, the severity of motor dysfunction increases [35]. This is a fact that was verified by results of the current study, where young female rats demonstrated severe motor impairment. This is in line with previous studies [6,36], which demonstrated that clioquinol induced symmetrical demyelination of lateral posterior funiculi of the spinal cord, optic nerve, and peripheral nerves.

Patients who suffered SMON, especially women, had significantly worse baseline scores characterized by enhanced anxiety and disturbed peace of mind [28]. During PCA analysis, the third component PCIII explained 22% of the total variation, and represented anxiety-related behavior. This is observed in clioquinol-treated patients suffering from irritability and neurosis by 27.8% and 13.6%, respectively [16], which is not as prevalent as sensory and motor abnormality. No previous studies elucidated the anxiogenic effect, but it may be related to N-methyl-D-aspartate receptor activation [28]. In the current experiment, DHQ reduced the OF central time that positively loaded on PCIII, which determined the anxiogenic effect of DHQ and influenced rats to leave the OF center arena in anxiety-like behavior.

The current study did not utilize different periods and pediatric age <P21 to study long-term effects of DHQ, where the consequences and the underlying physiopathological mechanisms need further investigation. As well, per the findings of the present study, another approach that should be investigated is the effect of DHQ on the microbiota–gut–brain axis and its effect on neuropsychiatric disorders, as demonstrated by Generoso et al. [37] and presented in a comparative study with another relevant antibiotic. The present study assessed the damage in dopaminergic pathway viz the nigrostriatal pathway, in addition to the anxiogenic tendency of DHQ; therefore, measuring the neurotransmitters in different brain areas could evolve the novel link between DHQ and neuropsychiatric disorders.

## 4. Materials and Methods

### 4.1. Animals

The study was conducted using young and adult Wistar rats of both sexes (3 weeks old, weighing 90–100 g and 4 months old, weighing 250–300 g, respectively). Animals were acquired from the Nile Company for Pharmaceuticals & Chemical Industries, Cairo, Egypt. They were allowed to acclimatize to laboratory conditions in the animal house of the Faculty of Pharmacy, Cairo University for 2 weeks before the experiment. They were kept under standard laboratory conditions for room temperature (24–26 °C), relative humidity (60 ± 10%), and light/dark cycle (12/12 h). Animals were allowed free access to standard chow diet and water. All procedures performed on animals were in accordance with the Guide for Care and Use of Laboratory Animals (NIH Publication No. 85-23, revised 2011) and was approved by the Ethical Committee of the Faculty of Pharmacy, Cairo University (Permit Number: PT 2732).

### 4.2. Drugs and Chemicals

Diiodohydroxyquinoline was acquired from CID-Chemical Industries Development (Cairo, Egypt). DHQ was suspended in 1% tween 80 saline solution. All other chemicals were of high purity and analytical grade.

### 4.3. Experimental Design

Eighty rats were randomly distributed using a computer-generated randomization table according to age and sex in eight groups (10 rats each). Animals in the first 4 groups received saline daily by oral gavage for 4 weeks and served as control. The control young male and female rats were allocated to group I (CYM) and group II (CYF), respectively. Control adult male and female rats were allocated to group III (CAM) and group IV (CAF), respectively, whereas, rats in the remaining 4 groups received DHQ daily by oral gavage for 4 weeks. Treated young male and female rats were allocated to group V (TYM) and group VI (TYF), respectively, and they received DHQ at a dose of 247.4 mg/kg/day. Treated adult male and female rats were allocated to group VII (TAM) and group VIII (TAF), respectively, and they received DHQ at a dose of 176.7 mg/kg/day. The doses of DHQ were converted from the maximum recommended daily human pediatric/adult doses [38,39] to rat doses using the conversion guide provided by Nair and Jacob [40]. Behavioral analysis was conducted on the last 2 days of the experiment in the following sequence: open field, Randall–Selitto, and hind paw cold allodynia tests on day 27, in addition to rotarod and hot plate tests on day 28. All behavioral testing was performed in a sound-isolated laboratory during the light phase, with a 2-h rest period between the tests [41]. Twenty-four hours after the completion of behavioral examination, rats were sacrificed by decapitation under light anesthesia. Brains, spinal cords, and sciatic nerves were quickly harvested and rinsed with ice-cold saline. They were fixed in 10% buffered formol saline for histopathological examination.

### 4.4. Behavioral Assessment

#### 4.4.1. Open Field Test

Open field test (OFT) is widely used to test exploratory behavior and general activity of rodents. The open field consisted of a square wooden box (80 × 80 × 40 cm) with red walls and white smooth polished floor divided by black lines into 16 equal squares (20 × 20 cm). Each rat was placed gently in the central area of the open field and allowed to freely explore the area for 3 min. The floor and walls were cleaned after testing each rat to eliminate possible bias due to odors left by previous rats. A video camera was fixed on the top of the box to record the movement and behavior of rats, which were analyzed using ANY-Maze video tracking software (Stoelting Co., Wood Dale, Illinois, USA). Total distance traveled (OFTD), mean speed (OFTMS), time immobile (OFTTI), central time (OFTCT), central distance (OFTTD), and thigmotaxis time (OFTTT) were recorded [42,43].

#### 4.4.2. Rotarod Test

The rotarod is a widely used to assess motor coordination and balance of rodents. Briefly, rats were trained for 2 consecutive days before the experimental procedures at a constant speed of 4 rpm on an automated 5-lane rotarod apparatus (Model 47750, Ugo Basile, Gemonio, VA, Italy) with 5-min cut-off time for 3 trials each day. The animals that stayed on the rod for 5 min were selected for experimentation. On the test day, rats were placed on the rod at a speed accelerated from 4 to 40 rpm with a 5-min cut-off time for 3 trials. The average rotarod fall off latency (RRFOL) was recorded for each rat [44].

#### 4.4.3. Randall–Selitto Test

Randall–Selitto test is used to quantify neuropathic pain response in rodents. The mechanical nociceptive threshold (RSMT) was assessed using a paw pressure analgesiometer (Model 7200, Ugo Basile, Italy), which applies a steadily increasing force to a rat’s left hind paw. Animals were held with soft cotton cloth in order to immobilize them for measuring the threshold. A withdrawal of the left hind paw or vocalization was considered as the endpoint. A cut-off pressure of 350 g was maintained to avoid tissue injury [45].

#### 4.4.4. Hot Plate Test

Hot plate test is used to assess heat thermal sensitivity of rats. Each rat was gently placed on the hot plate (Model 7280, Ugo Basile, Italy), which was set at 55 ± 1 °C and the time until either hind paw licking or hopping to avoid heat pain was recorded as hot plate reaction latency (HPRL), with a cut-off time of 20 s [46].

#### 4.4.5. Hind Paw Cold Allodynia Test

Cold allodynia test is used to assess cold thermal sensitivity of rats. The hind paws of each rat were immersed gently in a beaker containing ice-cold water maintained at 4 ± 1 °C. Then, the paw withdrawal latency (CAPWL) for each rat was determined. Only one hind paw was evaluated during each immersion at a time, with a cut-off time of 20 s. For each animal, two readings were taken for each hind paw at a 5-min interval, and CAPWL was calculated as the mean of both hind paw’s readings. The extended CAPWL was considered as antiallodynic effect, while the shorter CAPWL was interpreted as more severe allodynia [47].

### 4.5. Histological Assessment

Specimens from brain, spinal cord, and sciatic nerve were quickly harvested from 5 rats per each group and fixed in 10% buffered formol saline, embedded in paraffin, and then cut into 4 μm sections. Sections were deparaffinized, hydrated, and stained with Hematoxylin and Eosin (H&E) and Luxol fast blue (LFB) for examination under the light microscope (Olympus BX50, Olympus LS, Shinjuku-ku, Tokyo, Japan). The recorded histopathological lesions in H&E-stained sections from all rats were scored via assessing the percentage of lesions frequency from 5 randomly selected microscopic fields per animal using the following score system; 0 = absence of the lesions in all rats of the group, 1 = 1–10%, 2 = 11–25%, 3 = 26–50%, 4 = 51–75%, and 5 = over 75% [48]. In addition, the optical density of the LFB-stained sections was analyzed using image analysis software (Image J, version 1.46a, NIH, Bethesda, MD, USA).

### 4.6. Statistical Analysis

Data are expressed as mean ± S.D. Comparisons between means were performed using three-way analysis of variance (MANOVA) test, followed by Tukey’s multiple comparisons test using GraphPad Prism^®^ software package, version 8 (GraphPad Software Inc., San Diego, CA, USA). Correlations between behavioral measures were detected using Pearson’s correlation matrix (two-tailed). The level of significance was fixed at *p* < 0.05 for all statistical tests. To reduce the number of dimensions, a Factor analysis (principal component (PC) with Varimax rotation and Kaiser normalization, maximally 25 iterations to convergence) based on the correlation matrix was performed for all variables. The Varimax rotation was chosen because it is an orthogonal rotation method so that different factors do not intercorrelate; thus, each factor represents an independent behavioral pattern. Varimax rotation reduces the number of variables with high multiple factor loadings. Extracted components with eigenvalues higher than one were considered for further interpretation. Factor loadings >0.7 or <−0.7 were considered significant. Factor and correlation analyses were performed using SPSS Version 20.

## 5. Conclusions

Experimentally, sensory and motor functions, as well as extensive structural abnormalities in central and peripheral nervous tissues after the sub-acute administration of DHQ, were demonstrated; this finding calls for further investigation of the long-term use of this class of drugs, considering their benefit against alternative antibiotics. The study also highlights the role of gender and age in exacerbating quinoline-induced neurotoxicity. More preclinical studies are needed to study the demyelinating hazards of quinoline derivatives. In addition, it would be essential to study the effects of other factors, such as race and the microbiota–gut–brain interaction, which could affect the toxicity of the sub-acute administration of quinoline.

## Figures and Tables

**Table 1:** Pearson’s correlation matrix (correlation coefficient “r” and two-tailed *p*-value) between measured behavioral variables.

		OFTD	OFTMS	OFTTI	OFTCT	OFTCD	OFTTT	RRFOL	CAPWL	HPRL
**OFTMS**	**R** ***p***	**0.5927** **<0.0001**								
**OFTTI**	**r** ***p***	**−0.5052** **<0.0001**	**−0.4364** **0.0001**							
**OFTCT**	**r** ***p***	0.0374 0.7550	0.0111 0.9263	−0.1215 0.3092						
**OFTCD**	**r** ***p***	**0.3439** **0.0030**	0.1432 0.2300	**−0.3376** **0.0037**	**0.5176** **<0.0001**					
**OFTTT**	**r** ***p***	−0.1478 0.2151	−0.0662 0.5803	0.0859 0.4731	**−0.7676** **<0.0001**	**−0.4508** **<0.0001**				
**RRFOL**	**r** ***p***	**0.8741** **<0.0001**	**0.4706** **<0.0001**	**−0.5371** **<0.0001**	0.1480 0.2147	**0.4340** **0.0001**	**−0.2458** **0.0374**			
**CAPWL**	**r** ***p***	**0.3865** **0.0007**	0.1917 0.1067	**−0.3196** **0.0062**	**0.2710** **0.0213**	**0.2633** **0.0254**	**-0.3138** **0.0072**	**0.5724** **<0.0001**		
**HPRL**	**r** ***p***	**0.3547** **0.0022**	0.1485 0.2132	−0.2179 0.0659	**0.2537** **0.0315**	0.2198 0.0635	**−0.2766** **0.0186**	**0.5722** **<0.0001**	**0.8728** **<0.0001**	
**RSMT**	**r** ***p***	**0.2752** **0.0193**	0.1131 0.3441	−0.2083 0.0791	**0.2674** **0.0231**	**0.2327** **0.0491**	−0.2255 0.0567	**0.5419** **<0.0001**	**0.8355** **<0.0001**	**0.8662** **<0.0001**

Correlations with *p*-value <0.05 are highlighted in red. OFT; open field test, OFTD; distance traveled, OFTMS; mean speed, OFTTI; time immobile, OFTCT; central time, OFTCT; central distance, OFTTT; thigmotaxis time, RRFOL; rotarod test fall off latency, CAPWL; cold allodynia paw withdrawal latency, HPRL; hot plate test reaction latency, RSMT; Randall-Selitto mechanical threshold.

**Table 2:** Rotated (Varimax-Kaiser normalization) matrix of extracted components with eigenvalue > 1.

Variables	Component			Contribution
	1 (29.1%)	2 (27.4%)	3 (22.0%)	
**OFTD**	0.232	**0.878**	0.059	0.828
**OFTMS**	−0.013	**0.790**	−0.021	0.625
**OFTTI**	−0.107	**−0.728**	−0.128	0.558
**OFTCT**	0.150	−0.053	**0.913**	0.859
**OFTCD**	0.079	0.361	**0.700**	0.626
**OFTTT**	−0.163	−0.021	**−0.874**	0.791
**RRFOL**	0.484	**−0.771**	0.155	0.853
**CAPWL**	**0.901**	0.217	0.173	0.888
**HPRL**	**0.938**	0.154	0.131	0.920
**RSMT**	**0.932**	0.105	0.127	0.896

Percent values give the portion of explained variance for each factor. Significant factor loadings (>0.7, <−0.7) are given in bold. OFT; open field test, OFTD; distance traveled, OFTMS; mean speed, OFTTI; time immobile, OFTCT; central time, OFTCT; central distance, OFTTT; thigmotaxis time, RRFOL; rotarod test fall off latency, CAPWL; cold allodynia paw withdrawal latency, HPRL; hot plate test reaction latency, RSMT; Randall-Selitto mechanical threshold.

## Data Availability

Data is contained within the article and supplementary material.

## Supplementary Materials

The following are available online at https://www.mdpi.com/article/10.3390/ph15020251/s1, Table S1: Supplementary Table S1 Histopathological lesion scoring for cortex, striatum, spinal cord, and sciatic nerve in diiodohydroxyquinoline-treated animals.

## Abbreviations

OFT	Open field test
OFTD	Open field test distance
OFTMS	Open field test mean speed
OFTTI	Open field test time immobile
OFTCT	Open field test central time
OFTCD	Open field test central distance
OFTTT	Open field test thigmotaxis time
RRFOL	Rotarod fall off latency
CAPWL	Cold allodynia paw withdrawal latency
HPRL	Hotplate reaction latency
RSMT	Randall–Salyetto mechanical threshold
CYM	Control young male
CYF	Control young female
CAM	Control adult male
CAF	Control adult female
TYM	Treated young male
TYF	Treated young female
TAM	Treated adult male
TAF	Treated adult female
DHQ	Diiodohydroxyquinoline
8-HQ	8-hydroxyquinoline
SMON	Subacute myelo-optic neuropathy
PC	Principal component
TRPA1	Transient receptor potential ankyrin 1
H&E	Hematoxylin and Eosin
LFB	Luxol fast blue
MANOVA	Three-way analysis of variance test
PCA	Principal component analysis
