# Correction: Kamel et al. Experimental Evidence for Diiodohydroxyquinoline-Induced Neurotoxicity: Characterization of Age and Gender as Predisposing Factors. *Pharmaceuticals* 2022, *15*, 251

**DOI:** 10.3390/ph18050645

**Published:** 2025-04-28

**Authors:** Ahmed S. Kamel, Ahmed F. Mohamed, Mostafa A. Rabie, Marwa E. Elsherbiny, Kawkab A. Ahmed, Mahmoud M. Khattab, Noha F. Abdelkader

**Affiliations:** 1 Department of Pharmacology and Toxicology, Faculty of Pharmacy, Cairo University, Giza 11562, Egypt; ahmed.seifeldin@pharma.cu.edu.eg (A.S.K.); ahmed.fathi@pharma.cu.edu.eg (A.F.M.); mostafa.mohammed@pharma.cu.edu.eg (M.A.R.); mahmoud.khattab@pharma.cu.edu.eg (M.M.K.); 2 Department of Pharmacology and Toxicology, Faculty of Pharmacy, Ahram Canadian University, 6th of October City 12566, Egypt; marwae@ualberta.ca; 3 Department of Pathology, Faculty of Veterinary Medicine, Cairo University, Giza 12211, Egypt; kawkababdelaziz@yahoo.com

In the original publication [1], there was a mistake in Figure 6 as published. An unintended error during the insertion of photo b (spinal cord of control young female) in the panel of Figure 6 occurred. The corrected Figure 6 appears below. The authors state that the scientific conclusions are unaffected. This correction was approved by the Academic Editor. The original publication has also been updated. 
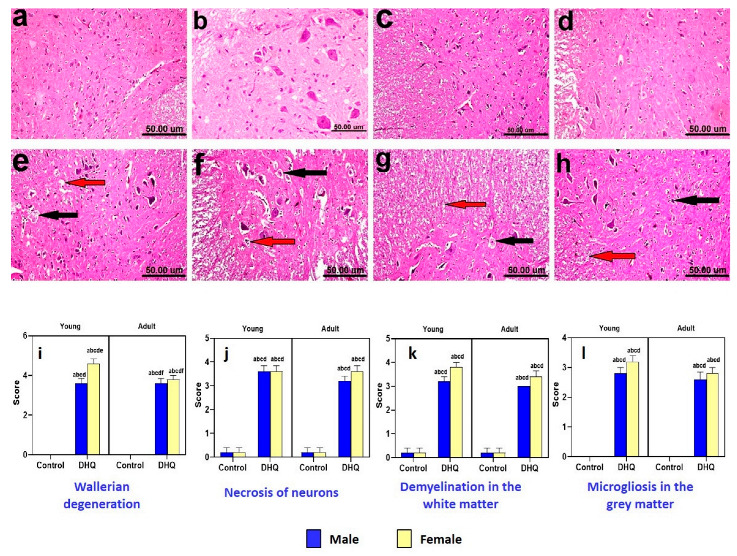


## References

[1] Kamel A.S., Mohamed A.F., Rabie M.A., Elsherbiny M.E., Ahmed K.A., Khattab M.M., Abdelkader N.F. (2022). Experimental Evidence for Diiodohydroxyquinoline-Induced Neurotoxicity: Characterization of Age and Gender as Predisposing Factors. Pharmaceuticals.

