# Supplementary material for: Experimental Evidence for Diiodohydroxyquinoline-Induced Neurotoxicity: Characterization of Age and Gender as Predisposing Factors
Source: Pharmaceuticals (Basel). 2022 Feb 19;15(2):251. doi: 10.3390/ph15020251 (PMC8879898; doi:10.3390/ph15020251)
Supplement: Supplementary file 1 [file pharmaceuticals-15-00251-s001.zip › pharmaceuticals-1576979-supplementary.pdf]

Supplementary Table S1 Histopathological lesion scoring for cortex, striatum, spinal cord, and sciatic nerve in diiodohydroxyquinoline-treated animals

Organ	Histopathological lesion	CYM	CYF	CAM	CAF	TYM	TYF	TAM	TAF
Cerebral cortex	degeneration and necrosis of neurons	0 (0-1)	0 (0-1)	0 (0-1)	0 (0-1)	4 (3-4) abcd	5 (4-5) abcde	3 (3-4) abcdf	4 (3-4) abcdf
	Neurono phagia	0 (0-0)	0 (0-0)	0 (0-0)	0 (0-0)	3 (3-4) abcd	4 (3-4) abcd	3 (3-3) abcdf	3 (3-4) abcd
	Neuro fibrillary tangles	0 (0-0)	0 (0-0)	0 (0-0)	0 (0-0)	3 (3-4) abcd	4 (4-5) abcde	3 (3-4) abcdf	4 (4-5) abcdeg
	Cerebral congestion	0 (0-0)	0 (0-0)	0 (0-0)	0 (0-0)	3 (3-4) abcd	4 (4-5) abcde	4 (3-4) abcd	3 (3-4) abcdf
	Meningeal congestion	0 (0-0)	0 (0-0)	0 (0-0)	0 (0-0)	4 (4-5) abcd	5 (4-5) abcd	4 (3-4) abcdf	4 (4-5) abcd

	Focal gliosis	0 (0-0)	0 (0-0)	0 (0-0)	0 (0-0)	4 (3-4) abcd	4 (4-5) abcd	3 (2-3) abcdef	4 (3-4) abcdg
	Demyelination of nerve fibers	0 (0-0)	0 (0-0)	0 (0-0)	0 (0-0)	4 (3-4) abcd	5 (4-5) abcd	3 (3-4) abcd	4 (3-4) abcd
Striatum	Shrunken and pyknotic neurons	0 (0-0)	0 (0-0)	0 (0-0)	0 (0-0)	3 (3-4) abcd	4 (4-5) abcde	4 (3-4) abcd	4 (3-4) abcd
	Demyelination	0 (0-1)	0 (0-1)	0 (0-1)	0 (0-1)	3 (3-4) abcd	4 (4-5) abcde	3 (3-4) abcdf	3 (3-4) abcdf
	Spongiosis	0 (0-0)	0 (0-0)	0 (0-0)	0 (0-0)	3 (3-4) abcd	4 (4-5) abcde	3 (3-4) abcdf	2 (2-3) abcdefg
	Diffuse gliosis	0 (0-0)	0 (0-0)	0 (0-0)	0 (0-0)	3 (3-4) abcd	3 (3-4) abcd	2 (2-3) abcdef	4 (3-4) abcdg

Spinal cord	Wallerian degeneration	0 (0-0)	0 (0-0)	0 (0-0)	0 (0-0)	4 (3-4) abcd	5 (4-5) abcde	4 (3-4) abcdf	4 (3-4) abcdf
	Necrosis of neurons	0 (0-1)	0 (0-1)	0 (0-1)	0 (0-1)	4 (3-4) abcd	4 (3-4) abcd	3 (3-4) abcd	4 (3-4) abcd
	Demyelination in the white matter	0 (0-1)	0 (0-1)	0 (0-1)	0 (0-1)	3 (3-4) abcd	4 (3-4) abcd	3 (3-3) abcd	3 (3-4) abcd
	Microgliosis in the grey matter	0 (0-0)	0 (0-0)	0 (0-0)	0 (0-0)	3 (2-3) abcd	3 (3-4) abcd	3 (2-3) abcd	3 (2-3) abcd
Sciatic nerve	Demyelination of nerve fibers	0 (0-1)	0 (0-1)	0 (0-1)	0 (0-1)	3 (3-4) abcd	4 (3-4) abcd	3 (3-4) abcd	4 (3-4) abcd

	Degeneration of myelinated nerve fibers	0 (0-0)	0 (0-0)	0 (0-0)	0 (0-0)	3 (3-4) abcd	4 (4-5) abcde	3 (3-4) abcdf	3 (3-4) abcdf

The scoring system was configured to: score 0 = absence of the lesion in all rats of the group, score 1 = 1–10%, score 2 = 11–25%, score 3 = 26–50%, score 4 = 51–75%, score 5 = over 75%. Data are expressed as median (min-max). a vs control young male, b vs control young female, c vs control adult male, d vs control adult female, e vs treated young male, f vs treated young female, g vs treated adult male using three-way ANOVA followed by Tukey's post hoc test; $p<0.05$. CYM; control young male, CYF; control young female, CAM; control adult male, CAF; control adult female, TYM; treated young male, TYF; treated young female, TAM; treated adult male, TAF; treated adult female.
